# Is daily physical activity affected by dynamic hyperinflation in adults with cystic fibrosis?

**DOI:** 10.1186/s12890-018-0623-7

**Published:** 2018-04-19

**Authors:** Daniela Savi, Marcello Di Paolo, Nicholas J. Simmonds, Chiara Pascucci, Serena Quattrucci, Paolo Palange

**Affiliations:** 1 Department of Public Health and Infectious Diseases, Adult Cystic Fibrosis Center, Sapienza University of Rome, 00185 Rome, Italy; 2 Cystic Fibrosis Unit, Bambino Gesù Children’s Hospital, Rome, Italy; 3 Department of Cystic Fibrosis, Royal Brompton Hospital and Imperial College, London, SW3 6NP UK; 4 Department of Experimental Medicine, Sapienza University of Rome, 00185 Rome, Italy; 5 Department of Pediatrics, Cystic Fibrosis Center, Sapienza University of Rome, 00185 Rome, Italy; 6 Eleonora Lorrillard-Spencer Cenci Foundation, 00185 Rome, Italy

**Keywords:** Cystic fibrosis, Daily physical activity, Dynamic hyperinflation, Exercise intolerance

## Abstract

**Background:**

The aim of this study was to investigate the relationship between dynamic hyperinflation and daily physical activity (DPA) in adults with cystic fibrosis (CF).

**Methods:**

Thirty-four clinically stable CF were studied. All patients undertook incremental cardiopulmonary exercise testing (CPET). CPET-related measurements included: oxygen uptake (V’O_2_), carbon dioxide production (V’CO_2_), ventilatory profile, work rate (W), inspiratory capacity (IC), end-expiratory lung volume (EELV). PA was assessed using the accelerometer SenseWear Pro3 Armband.

**Results:**

Exercise tolerance was reduced in most of patients and the mean V’O_2,peak_ value was 75.2% of predicted (28.5 ± 4.8 ml/min/kg). Seventy % of patients responded to CPET with dynamic hyperinflation. Higher incidence of dynamic hyperinflation was found in CF males compared to CF females (*p* = 0.026). Patients who developed dynamic hyperinflation during CPET had higher vigorous PA (*p* = 0.01) and more total energy expenditure (*p* = 0.006) than patients who did not. EELVΔ was related to activities requiring vigorous intensity and total energy expenditure (*R* = 0.46, *p* = 0.001; *R* = 0.57, *p* <  0.001).

**Conclusions:**

In adults with CF and mild to moderate lung impairment, DPA might not be limited by dynamic hyperinflation.

**Electronic supplementary material:**

The online version of this article (10.1186/s12890-018-0623-7) contains supplementary material, which is available to authorized users.

## Background

The assessment of daily physical activity (DPA) is a parameter of increasing interest in the clinical evaluation of patients with cystic fibrosis (CF). Despite the well-recognized physical and psychological benefits of exercise in maintaining health in CF, exercise participation is often below recommended levels [1] especially in CF females [2]. Recently, two studies showed that activity levels in daily life correlate significantly with exercise tolerance in CF adults [3, 4]. It is important to identify significant factors related to aerobic capacity because peak oxygen uptake (V’O_2,peak_) is a prognostic factor in CF [5, 6]. Maintaining high levels of physical activity is an important objective in the management of CF.

Many studies have investigated exercise intolerance in CF and have showed multiple compromised mechanisms [7]. These include musculoskeletal abnormalities [8, 9], abnormal oxygen delivery and gas exchange [10], deconditioning [11] and finally lung dynamic hyperinflation [12]. In chronic obstructive pulmonary disease (COPD) breathing at higher lung volumes increases respiratory work and thus potentiates the perception of breathlessness, which may discourage patients from taking part in DPA and become responsible for a decrease in DPA [13].

In the past, Alison et al. measured changes in end-expiratory lung volume (EELV) during leg and arm exercise in 22 CF patients [14]. They observed that CF patients with FEV1 < 90% predicted were likely to dynamically hyperinflate during arm cranking. This is particularly interesting if we consider that the ability to perform upper-limb exercise is important for daily activities. More recently, a larger study showed that dynamic hyperinflation during cardiopulmonary exercise testing (CPET) is almost common in adult patients with mild-to-moderate CF [12]. To date, although the impact of dynamic hyperinflation on exercise is recognized [12], little information on the effect of dynamic hyperinflation on DPA is available in CF. Several studies have found that the level of DPA is independent of lung function in CF [15, 16] suggesting that high activity levels do not necessarily reflect the level of lung function [16]. Whether DPA level in adult CF can be associated with a development of dynamic hyperinflation warrants further investigations.

The aim of the present study was to investigate the relationship between dynamic hyperinflation developed during CPET and DPA levels in a cohort of adults with mild-to-moderate airflow obstruction. Specifically, we wished to evaluate if the identification of dynamic hyperinflation with exercise may correlate with daily life activities in these CF patients.

## Methods

### Study design

Thirty-four adults with CF were recruited consecutively between January 2012 and December 2013 from the Policlinico Umberto I Hospital, Sapienza University of Rome, Italy. The study was approved by the Ethics Committee of Policlinico Umberto I Hospital, with approval number 582/11. Data on demographic and clinical characteristics, baseline spirometry and CPET-related measurements were collected at the time of enrolment. The patients were equipped with a physical activity monitor. Their habitual physical activities were assessed for a period of 5 days. Medical management of the CF patients was carried out as normal, including use of bronchodilators. Subjects with CF were asked to continue any respiratory-related medications before the visits. The estimation of the calculation for sample size was done using a previous study by Lahaije Anke JMC, et al. [17] who showed a *r* = 0.42 for the relation between dynamic hyperinflation and physical activity. For this study, assuming α = 0.05 and 0.80 power with r between 0.40 and 0.50, our effect size would require a number of patients between 30 and 47. We therefore recruited 34 CF patients in total.

### Patients and data collection

Patients attending a CF outpatient clinic were approached for participation in the study. Patients were included if they were ≥ 18 years of age, with mild-to-moderate pulmonary impairment based on FEV_1_ (i.e., mild = FEV_1_ > 80% predicted; moderate = FEV_1_ 40 to 80% predicted) and had a confirmed diagnosis of CF based on genetic testing showing two CF-causing mutations and/or two documented sweet chloride values > 60 mEq/l. Patients were excluded if they had unstable medical conditions that could cause or contribute to breathlessness (i.e cardiovascular, metabolic, or other respiratory diseases) or other disorders that could interfere with exercise testing, such as neuromuscular diseases or musculoskeletal problems. Patients with a pulmonary exacerbation in the 4 weeks prior to the study, with acute respiratory failure, on oxygen therapy, on the waiting list for lung transplantation or post- transplant were excluded from the study. Baseline data were collected at the time of study entry.

After obtaining written informed consent and appropriate screening of medical history, we collected data on age, sex, height, weight, body mass index (BMI), chronic infections and CF comorbidity (pancreas insufficiency and CF-related diabetes). At the time of study entry all patients underwent spirometry, nitrogen washout and single-breath diffusing capacity (DLCO) which were performed by standardized techniques using an automated pulmonary function testing system (COSMED PFT, Pavona Italy) [18–20]. All pulmonary function data were standardized as percentages of predicted normal values [21, 22]. Maximal voluntary ventilation (MVV) was estimated using the formula FEV_1_ × 40.

A maximal incremental CPET was conducted. We used an electronically-braked cycle ergometer (Ergoline-800, Ergoline GmbH, Bitz, Germany) and the Quark b^2^ system (COSMED, Rome, Italy) according to recommended guidelines [23]. CPET consisted of a steady-state resting period, then 2 min of warm-up at 10 watt followed by a stepwise protocol in which the work rate was increased in 1-min intervals by increments of 20 W. The test was continued until exhaustion and subjects were encouraged throughout the test. Subjects rated the magnitude of their perceived breathing and leg discomfort at rest, every two minutes during exercise and at peak exercise by pointing a modified Borg scale [24]. Oxygen saturation (SpO_2_) by pulse oximetry on the finger, electrocardiographic monitoring of heart rate (HR), rhythm and ST-segment changes, minute ventilation (V’E), oxygen uptake (V’O_2_), carbon dioxide production (V’CO_2_) were measured at rest and during CPET. Peak V’O_2_ (V’O_2,peak)_ was normalized for body weight and was also expressed as percentage of the predicted value [25]. The inspiratory capacity (IC) maneuver was performed by patients at rest, at the end of each increment of exercise and at peak of exercise. The IC represents the volume inhaled from the end of normal exhalation to maximal inhalation (i.e., total lung capacity). EELV was calculated by subtracting the IC from the total lung capacity (EELV = TLC-IC) [26]. It is assumed that total lung capacity remains unchanged during exercise, which is supported by literature in health [27] and COPD [28]. The change in the IC from rest to peak exercise was calculated (ICΔ) and consequently, the change in EELV from rest to peak exercise was also calculated (EELVΔ). Based on previous studies in CF [12, 29], patients were categorized into the dynamic hyperinflation group if they showed evidence of decreasing in IC from rest to peak exercise (ICΔ) ≥ 100 mL.

### Assessment of daily physical activity

DPA was assessed at the time of the study enrollment, using a multi-sensor armband (SenseWear Pro3 Armband (SWA), BodyMedia, Pittsburgh, USA) which has been validated in CF [30, 31]. Patients wore the armband for at least five full consecutive typical days (including 3 weekdays and 2 weekend days) when they were at home. Data are reported as the average of 5 days. It was reported in CF that 5 days monitoring was enough to assess DPA and that DPA levels were similar through the week (i.e., weekdays versus weekend days) [3, 4]. The characteristics of the device have been previously described [2]. The outputs obtained from the armband were the detection of energy expenditure (EE), including total energy expenditure (TEE) and active energy expenditure (AEE), total physical activity duration, number of steps, time lying down, sleep duration and intensity of PA, expressed in metabolic equivalents (METS). The time (min) spent in PA at different intensities (mild, moderate, vigorous) and the definitions for activity levels based on METS were those used by Troosters et al. [3]. See Additional file 1.

### Statistical analysis

Data obtained from CF patients with dynamic hyperinflation and non-dynamic hyperinflation were compared. Categorical data are presented as percentages, and comparisons were performed using the *χ*^*2*^ or *Fisher’s exact test*. Parametric data are presented as mean ± standard deviation (SD) and comparisons were made using the two-sample independent *t test*. Non-parametric data are presented as median and interquartile range and comparisons were performed using the *Mann–Whitney U test*. Correlations were identified between PA measurements and CPET parameters through the use of the *Pearson’s R correlation coefficient* or the *Spearman’s rank correlation coefficient* according to parametric or non-parametric distribution of data, respectively and corrected for gender. Significant contributors (gender, V’O_2,peak_ ml∙min^− 1^, EELVΔ in absolute value and % of TLC) were introduced into a stepwise multiple linear regression analysis to identify independent determinants of PA.

All statistical tests were two-sided, and significance was reported at *p* <  0.05. Analyses were performed using the SPSS Statistics version 22.0 software package (IBM, Armonk, New York, USA).

## Results

### Study population characteristics and daily physical activity

Baseline characteristics and pulmonary function data for the group as a whole and the dynamic (*n* = 24) and non-dynamic hyperinflation (*n* = 10) groups are shown in Table 1. The study group comprised CF patients with a good nutritional status and a mild to moderate lung impairment based on FEV1 as a percentage of predicted. (Table 1). Seventy percent (24/34) of patients demonstrated evidence of dynamic hyperinflation during CPET. There was no difference for age, BMI, airways infection, pulmonary function and oxygen saturation between the dynamic hyperinflation and non-dynamic hyperinflation groups. However, the frequency distribution of males in the dynamic hyperinflation group was significantly greater than females (*p* <  0.05).

**Table 1:** Demographic characteristics and pulmonary function of the study group

Characteristics	All (*n* = 34)	Dynamic hyperinflation (*n* = 24)	Non-dynamic hyperinflation (*n* = 10)	*p* value
Age, years	33.1 ± 8.5	34.2 ± 9.0	30.5 ± 6.7	0.322
BMI, kg/m^2^	22.6 ± 2.6	23.0 ± 2.3	21.5 ± 3.0	0.093
Male, %	67	79	40	0.026
Pseudomonas aeruginosa infection, %	64.7	70.8	50.0	0.247
*Staphylococcus aureus* infection, %	67.6	62.5	80.0	0.320
Burkholderia cepacia infection, %	2.9	4.2	0	0.512
Pancreatic insufficiency, %	73.5	75.0	70.0	0.763
F508 del homozygous/heterozygous	6/21	5/14	1/7	0.731
Lung function				
FEV_1_, % of predicted	69.6 ± 19.0	68.3 ± 19.2	72.9 ± 19.3	0.532
FVC, % of predicted	86.1 ± 17.2	85.9 ± 17.6	86.5 ± 17.2	0.921
FEV_1_/FVC, %	67.9 ± 10.9	63.3 ± 10.7	71.8 ± 11.1	0.189
IC, % of predicted	100.8 ± 23.3	102.8 ± 24.2	96.1 ± 21.9	0.478
TLC, % of predicted	95.6 ± 15.9	96.9 ± 17.4	92.5 ± 11.9	0.473
RV, % of predicted	112.2 ± 45.2	117.9 ± 51.0	98.6 ± 23.7	0.183
RV/TLC,%	31.6 ± 9.4	32.4 ± 10.0	29.8 ± 8.3	0.483
FRC, % of predicted	93.5 ± 27.5	95.7 ± 31.9	88.5 ± 12.9	0.859
DL_CO_, % of predicted	81.3 ± 13.7	82.1 ± 13.2	79.1 ± 15.7	0.612
MVV, l·min^− 1^	104.6 ± 31.3	107.5 ± 32.5	97.4 ± 28.3	0.396
SpO_2_, %	96.8 ± 1.6	96.7 ± 1.7	97.2 ± 1.3	0.445

*Definition of abbreviations*: *BMI* body mass index, *FEV*_*1*_ forced expiratory volume in one second, *FVC* forced vital capacity, *FEV*_*1*_*/FVC* forced expiratory volume in one second and forced vital capacity ratio, *TLC* total lung capacity, *RV* residual volume, *IC* inspiratory capacity, *FRC* functional residual capacity, *DL*_*CO*_ diffusion lung capacity for carbon monoxide, *MVV* estimated maximal voluntary ventilation, *SpO*_*2*_ arterial oxygen saturation, *% of predicted* percentage of predicted normal values

Data are presented as mean ± SD. *p* values are differences between the dynamic hyperinflation and non-dynamic hyperinflation groups

Physiological responses to CPET in the dynamic and non-dynamic hyperinflation groups are presented in Table 2. Exercise tolerance was reduced in most of patients. Their mean V’O_2,peak_ value was 75.2% of predicted (28.5 ± 4.8 ml/min/kg). The majority of CF patients (24 of 34) increased their EELV back toward resting levels at maximal exercise and did increase EELV beyond resting values, demonstrating evidence of dynamic hyperinflation during CPET. In the dynamic hyperinflation group, the IC from rest to peak exercise decreased by 0.5 ± 0.3 l as showed in Table 3 by EELVΔ value.

**Table 2:** Daily physical activity of the study group

Variable	All (*n* = 34)	Dynamic hyperinflation (*n* = 24)	Non-dynamic hyperinflation (*n* = 10)	*p* value
Total energy expenditure, kcal	2577 (2395–3014)	2785 (2513–3093)	2260 (2018–2541)	0.006
Active Energy expenditure, kcal	695 (497–1327)	744 (541–1516)	580 (376–1044)	0.109
Duration Physical Activity, min/day	179 (116–310)	187 (111–321)	168 (116–313)	0.849
Average METs, kcal·kg^− 1^·h^−1^	1.8 (1.6–2.0)	1.8 (1.6–2.0)	1.7 (1.6–2.0)	0.994
Steps, number/day	8784 (6533–11,321)	8784 (7031–11,763)	8647 (5172–11,427)	0.624
Mild intensity activities, min/day	160 (106–253)	167 (93–268)	151 (106–287)	0.944
Moderate intensity activities, min/day	14 (9–29)	15 (9–32)	12 (5–24)	0.270
Vigorous intensity activities, min/day	1 (0–3)	1 (1–4)	0	0.010
Moderate+Vigorous intensity activities, min/day	16 (9–29)	16 (10–35)	12 (5–24)	0.196

*Definition of abbreviations*: *CF* Cystic Fibrosis, *METs* Metabolic Equivalents of Task

Data are presented as median (interquartile range). *p* values are differences between the dynamic hyperinflation and non-dynamic hyperinflation groups

**Table 3:** Cardiopulmonary exercise testing data of the study group

CPET variables at peak exercise	All (*n* = 34)	Dynamic hyperinflation (*n* = 24)	Non-dynamic hyperinflation (*n* = 10)	*p* value
Work rate, % of predicted maximum	87.5 ± 18.4	85.2 ± 14.7	93.2 ± 25.2	0.365
V’O_2_, ml∙min^−1^∙kg^− 1^	28.5 ± 4.8	28.7 ± 4.6	27.8 ± 5.6	0.639
V’O_2_, % of predicted maximum	75.2 ± 13.3	75.2 ± 13.5	75.0 ± 13.4	0.960
HR, beats∙min^− 1^	155.4 ± 12.4	155.0 ± 12.5	156.3 ± 12.6	0.785
HR, % of predicted maximum	83.3 ± 7.4	83.6 ± 7.9	82.5 ± 6.4	0.697
V’O_2_/HR, ml∙beat^− 1^	12.2 ± 3.1	12.9 ± 3.0	10.5 ± 2.5	0.037
SpO_2_, %	93.6 ± 3.3	93.2 ± 3.4	94.7 ± 3.0	0.254
SpO_2_Δ, %	−3.2 ± 3.2	−3.5 ± 3.3	−2.5 ± 2.8	0.467
V_T_, l	1.9 ± 0.6	2.0 ± 0.5	1.6 ± 0.6	0.040
Respiratory rate, breaths∙min^− 1^	37.8 ± 7.6	37.4 ± 6.7	38.8 ± 10.0	0.655
V’E, l∙min^− 1^	68.8 ± 19.9	73.2 ± 19.1	58.2 ± 18.7	0.042
BR, %	31.2 ± 19.6	28.8 ± 18.7	36.7 ± 21.6	0.292
V’E/V’CO_2_ slope	27.4 ± 5.3	28.2 ± 5.9	25.5 ± 2.8	0.181
PET_CO2_, mmHg	40.1 ± 5.5	39.5 ± 5.8	41.3 ± 4.8	0.304
EELV, l	3.8 ± 1.4	4.3 ± 1.2	2.7 ± 1.3	0.001
EELVΔ, l	0.2 ± 0.6	0.5 ± 0.3	− 0.5 ± 0.6	< 0.001
EELVΔ, % of TLC	2.6 ± 12.5	8.0 ± 5.4	− 10.4 ± 15.3	< 0.001
Dyspnea, modified Borg scale	4.7 ± 2.5	5.1 ± 2.5	4.0 ± 2.4	0.183
Leg discomfort, modified Borg scale	6.3 ± 2.1	6.6 ± 2.2	5.6 ± 1.8	0.223

*Definition of abbreviations*: *CF* Cystic Fibrosis, *V’O*_*2*_ oxygen uptake, *HR* heart rate, *SpO*_*2*_ arterial oxygen saturation, *V*_*T*_ tidal volume, *V’E* minute ventilation, *BR* breathing reserve, *V’E/V’CO*_*2*_ ventilatory equivalent for carbon dioxide, *PET*_*CO2*_ partial pressure of end-tidal CO_2_, *EELV* end-expiratory lung volume, *EELVΔ* end-expiratory lung volume delta from rest to peak exercise, *TLC* total lung capacity

Data recorded at peak exercise. Data are presented as mean ± SD, unless otherwise stated. *p* values are differences between the dynamic hyperinflation and non-dynamic hyperinflation groups

When we analyzed dynamic hyperinflation at submaximal level of exercise (i.e. at lactic threshold, LT), there was no difference for EELVΔ value between hyperinflated and not hypeinflated CF patients (− 0.09 ± 0.22 l versus − 0.11 ± 0.09 respectively, *p* = 0.50).

Patients with evidence of dynamic hyperinflation had similar V’O_2_, work-rate and heart rate at peak exercise to those without evidence of dynamic hyperinflation. Both groups achieved maximal heart rates above 80% of predicted. In dynamic hyperinflation group we found higher values of VˈE_peak_ (*p* <  0.05) and lower values of breathing reserve (BR) even if did not achieve the statistical significance (*p* = 0.2). For both groups, mean maximal ventilation was less than the predicted MVV and breathing reserve was always preserved, suggesting that ventilation limit was not a main limiting factor. Both CF groups stopped exercise primarily because of leg discomfort. No differences were found in the ventilatory equivalents for carbon dioxide (V’E/V’CO_2_) at peak exercise; no differences were detected for V’E/V’CO_2_ slope and PETCO_2_, indicating similar ventilatory efficiency and gas exchange functionality. There was a fall in mean SpO_2_ from rest to peak exercise (SpO_2_Δ) in hyperinflated patients (− 3.5%) which exceeded that seen in non-hyperinflated patients (− 2.5%) but was not statically different.

DPA is shown in Table 3. Patients with evidence of dynamic hyperinflation demonstrated higher total energy expenditure and higher daily time spent in activities above vigorous intensity. Similarly, there was a trend for more activities above moderate intensity (*p* = 0.1) and active energy expenditure (*p* = 0.09) in the dynamic hyperinflation group.

**Fig. 1:**
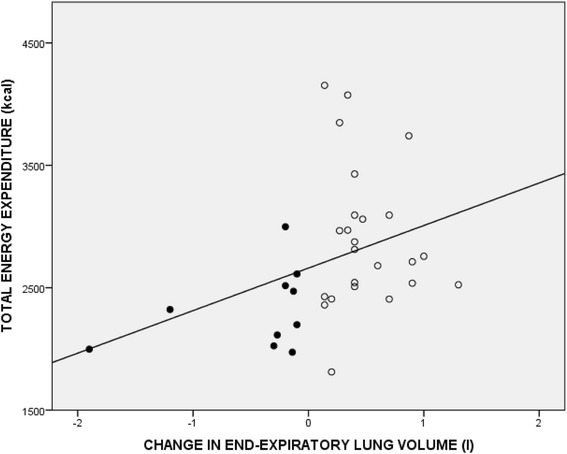
Relationship between total energy expenditure in daily living recorded by accelerometer and change in end-expiratory lung volume (EELV) in patients with cystic fibrosis (*R* = 0.57, *p* < 0.001). In this figure, the most hyperinflated CF patients, those with the highest EELV, also showed the highest physical activity parameter represented as total energy expenditure. Open circles = CF patients with dynamic hyperinflation, closed circles = CF patients without dynamic hyperinflation

Correlation analysis was done for the group as a whole. DPA above moderate intensity was related to V’O_2,peak_ expressed as ml∙min^− 1^ (*R* = 0.44, *p* = 0.002) and as ml∙min^− 1^∙kg^− 1^ (*R* = 0.52, *p* < 0.001). Interestingly, daily activity measures were related to the development of dynamic hyperinflation. We found a relationship between total energy expenditure and EELV_peak_ (*R* = 0.56, *p* < 0.001)_,_ EELV_peak_ expressed as % of TLC (*R* = − 0.47, *p* = 0.001). Moreover, we found a positive relationship between total energy expenditure and change in EELV from rest to peak exercise (EELVΔ) (*R* = 0.57, p < 0.001, Fig. 1). A relationship was also found between activities requiring vigorous intensity and EELV_peak_ (*R* = 0.59, *p* < 0.001) and EELVΔ (*R* = 0.46, *p* = 0.001).

Among these significant contributors to DPA, the multivariate linear regression analysis identified V’O_2,peak_ and gender as independent predictor of DPA and excluded the role of dynamic hyperinflation in predicting DPA levels (Table 4). Specifically, EELVΔ (l) and EELVΔ (% of TLC) were discarded as not significant predictors: EELVΔ (l) *p* values were 0.412, 0.513 and 0.719 for model 1, 2 and 3, respectively; EELVΔ (% of TLC) *p* values were 0.796, 0.581 and 0.691 for model 1, 2 and 3, respectively (Table 4).

**Table 4:** Independent predictors of physical activity level in a multivariate linear regression analysis

Model	Dependent	Predictors	Unstandardized coefficients		Standardized coefficients	95% CI	p	R^2^
			T	SE	β			
1	Total Energy Expenditure	(Constant)	1582.144	302.285		966.334 to 2197.955	< 0.001	
		Gender	450.965	184.121	0.412	75.877 to 826.054	0.020	
		V’O2 peak	0.450	0.200	0.378	0.042 to 0.857	0.032	
								52.9%
2	Vigorous PA	(Constant)	0.091	0.523		−0.973 to 1.155	0.863	
		Gender	3.040	0.856	0.526	1.297 to 4.782	0.001	
								27.7%
3	Moderate + Vigorous PA	(Constant)	−11.567	11.908		−35.797 to 12.663	0.338	
		VˈO2 peak	0.018	0.007	0.418	0.004 to 0.032	0.013	
								17.5%

*Definition of abbreviations*: *CF* Cystic Fibrosis, *V’O*_*2*_ oxygen uptake, *PA* physical activity, *CI* confidence interval. For every model: included independent variables were: gender (male), V’O2 peak (ml∙min^−1^), EELVΔ (l) and EELVΔ (% of TLC)

For every model: EELVΔ (l) and EELVΔ (% of TLC) were discarded as not significant predictors. For model 2 and 3: V’O2 peak (ml∙min^− 1^) and gender (male) were discarded as not significant predictors, respectively

## Discussion

The main finding of this study is that DPA levels of patients with mild-to moderate CF might not be correlated with the development of dynamic hyperinflation. Specifically, the variance in DPA within these CF patients cannot be explained by the dynamic hyperinflation that occurs during CPET. Patients who responded to exercise with dynamic hyperinflation performed higher daily activity requiring vigorous intensity and had more total energy expenditure than those without dynamic hyperinflation.

It is well known that the development of dynamic hyperinflation makes patients breathe at increased operational lung volumes, resulting in a higher respiratory effort and oxygen requirement of breathing [32]. Dynamic hyperinflation leaves less room for the expansion of tidal volume that comes with increasing minute ventilation. Several studies are available for COPD patients, which observed that dynamic hyperinflation is likely to lead to such mechanical constraints on tidal volume [26, 33]. At some point, further increase in the effort to breathe does not result in an equal increase in tidal volume, known as neuromechanical dissociation [33]. This causes an increase in symptoms and impairments that patients experience [33]. As consequence, in moderate-severe COPD patients, reduced DPA might be partially explained by the development of dynamic hyperinflation during CPET [13]. Although extrapolation of data from COPD to CF is not appropriate, the COPD model is used to explain some physiological mechanisms during exercise mainly because the study of dynamic hyperinflation and its clinical utility in CF is limited. Specifically, the aspects of lung mechanics related to daily activity have not been studied in detail in patients with CF, who are younger and less likely to be smokers [34]. In our mild-to moderate CF who developed dynamic hyperinflation during exercise, this neuromechanical dissociation did not happen, because their IC value at peak exercise was still large. We found that dynamic hyperinflation did not limit exercise tolerance on CPET in this CF group examined. The hyperinflated group reported symptoms of muscle effort equal or in excess of dyspnea. One explanation could be that the occurrence of hyperinflation during CPET played a minor role in limiting exercise tolerance in our CF patients, likely because of mild lung function impairment. This is in keeping with Moorcroft et al.’ conclusions that non pulmonary factors predominate in limiting patients with mild to moderate disease [35].

We were able to demonstrate a positive correlation between changes in EELV from rest to peak exercise and levels of DPA, expressed both as energy expenditure and activities requiring vigorous intensity. These features described that CF patients with the highest EELV also showed the highest physical activity parameters, suggesting that DPA might not be limited by the occurrence of dynamic hyperinflation. There are different procedures to estimate DPA using accelerometers. One of the most common is estimating the energy expenditure of the activity performed. Several studies have highlighted the accuracy of SWA on estimate energy expenditure in CF. It was found an agreement between energy expenditure measures estimated by the SWA and indirect calorimetry during daily activities [31]. Furthermore, the hypersalinity of sweat in people with CF had no significant negative impact on the accuracy of SWA estimate of energy expenditure [30].

In our patients, the multiple regression analysis did not identify dynamic hyperinflation as an independent variable. Aerobic capacity and gender were found significant predictor of DPA, explaining almost 53% of the variance in DPA of our patients with mild-to moderate lung disease. The DPA that a subject does throughout her/his habitual life can be influenced by many aspects. Except physical capabilities, whether a lifestyle is less or more active is determined by social, behavioural and psychological factors. We were also able to confirm previous results describing that the level of physical activity was independently related to gender in CF population [2, 16]. Recently, Stevens et al. studied for the first time the prevalence of dynamic hyperinflation in a large CF population with a broad range of lung disease severity [12]. The authors demonstrated that dynamic hyperinflation during CPET is common among CF patients with mild-to moderate lung impairment. In our study, we confirmed the fact that CF patients are likely to hyperinflate during exercise and our EELV values were comparable to those of Stevens et al. [12] (EELVΔ: 0.5 ± 0.3 l in our study vs. 0.44 ± 0.26 in Stevens’ study). They also showed that patients who developed dynamic hyperinflation had lower lung function, lower exercise tolerance and greater breathlessness at peak exercise than patients without dynamic hyperinflation. By contrast, we found no significant differences on aerobic capacity between CF patients with evidence of dynamic hyperinflation and patients without hyperinflation. One explanation could be that, in both studies, there was a greater gender difference on the distribution of males versus females in hyperinflated or not hyperinflated CF group. This may have possibly affected the exercise testing parameters. Specifically, in Stevens’s study the frequency distribution of males in the non-dynamic hyperinflation group was significantly greater than females (88%, *p* < 0.05) while in our study was significantly lower (40%, *p* = 0.02) [12]. For comparison, we retained that it would be more appropriate to scale the values for % of predicted and then analyse if there are differences between hyperinflated and not hypeinflated CF patients. Conversely, Stevens et al. presented their CPET results as absolute values rather than percentage of predicted [12]. The lower CPET parameters observed in their dynamic hyperinflated group might have been influenced by the fact that subjects were almost CF females and that the non-dynamic hyperinflation group was composed mainly by males. In this kind of gender distribution, we retain that should not be used the absolute values for CPET parameters to observe differences between dynamic hyperinflation and non-dynamic hyperinflation groups, because these parameters depend strongly on anthropometric features (i.e., height and sex) [25].

Following up on these results, the present study now confirms the high prevalence of dynamic hyperinflation in CF adult patients and adds that dynamic hyperinflation may not influence mild CF from taking part in DPA.

There is evidence that submaximal exercise related data (also known as lactate threshold-LT measures) are more reflective of exercise fitness [36] and would be more appropriate for comparison with DPA in order to investigate the possible effects of the latter on exercise tolerance. When we analyzed the EELVΔ value at submaximal level of exercise (i.e. at LT), which in turn should reflect more closely normal daily activities, there was no difference for EELVΔ value between hyperinflated and not hypeinflated CF patients. It seems that in patients with mild to moderate lung obstruction, dynamic hyperinflation started during an incremental CPET where the intensity of exercise become closer to the zone/range of the maximal effort. So, if we consider that activities of daily life are of sub-maximal intensity, they are also likely not to be limited by dynamic hyperinflation.

We recognize that detecting the occurrence of dynamic hyperinflation during daily life activities is important, but may be unfeasible in clinical practice. However, this can be determined alternatively during CPET. More studies may be interesting to evaluate the role of dynamic hyperinflation induced by exercise on daily activities, especially in CF patients with more advanced disease. In agreement with Stevens’ comment [12], we would expect a greater incidence of dynamic hyperinflation during exercise in patients with severe CF (i.e., FEV_1_ < 30%pred).

The study has important limitations. Firstly, the cohort is small and from a single centre so it has limited generalizability to the whole CF population. Secondly, more males than females were recruited. This was unintentional and a result of patient enthusiasm to participate at the study, but we recognise this may affect the interpretation of the results. Finally, because it is a cross-sectional study, it cannot establish a causal relationship, but instead an association between dynamic hyperinflation and PA.

## Conclusion

In CF patients with mild to moderate lung impairment, dynamic hyperinflation during exercise is very common and seems not to be a limiting factor for daily physical activity. More research is needed to study directly the development of dynamic hyperinflation during daily life activities and to establish whether hyperinflation could influence habitual PA in more advanced CF disease.

## Additional file

Additional file 1: Assessment of daily physical activity. Characteristics of the multi-sensor armband (SenseWear Pro3 Armband). Definitions for activity levels. (DOCX 16 kb)

## Abbreviations

BMI: Body mass index
BR: Breathing reserve
CF: Cystic Fibrosis
EELV: End-expiratory lung volume
FEV1: Forced expiratory volume in 1 s
FVC: Forced vital capacity
IC: Inspiratory capacity
PA: Physical activity
PET_CO2_: Partial pressure of end-tidal CO_2_
SpO_2_: Arterial oxygen saturation
TLC: Total lung capacity
V’E: Minute ventilation
V’E/V’CO_2_: Ventilatory equivalent for carbon dioxide
VO_2_: Oxygen uptake
V_T_: Tidal volume
