# Supplementary material for: Is daily physical activity affected by dynamic hyperinflation in adults with cystic fibrosis?
Source: BMC Pulm Med. 2018 Apr 19;18:60. doi: 10.1186/s12890-018-0623-7 (PMC5907710; doi:10.1186/s12890-018-0623-7)
Supplement: Supplementary file 1 — Assessment of daily physical activity. Characteristics of the multi-sensor armband (SenseWear Pro3 Armband). Definitions for activity levels. (DOCX 16 kb) [file 12890_2018_623_MOESM1_ESM.docx]

Additional file 1

**Methods**

Assessment of daily physical activity

The device was positioned on the upper right arm over the triceps muscle at the midpoint between the acromion and olecranon processes, as recommended by the manufacturer. Patients were instructed to wear the armband day and night and only to remove it for bathing or showering. Patients were also asked to continue any respiratory-related medications and any of their normal activities. The sensor contains a biaxial accelerometer, a galvanic skin response sensor, a heat flux sensor, a skin temperature sensor and a near-body ambient temperature sensor from which the data were stored minute by minute. Using specific software (v6.1) these variables, as well as body weight, height, handedness and smoking status (smoker or non-smoker), were used to estimate energy expenditure (EE). The outputs obtained from the armband was the time spent in physical activity at different intensities. The time (min) spent with an energy expenditure of 3-4.8 METS was considered “mild” activity (e.g., walking at normal walking speed, carrying out light household work), time spent at 4.8-7.2 METS was considered “moderate” activity (e.g., brisk walking or cycling) and activities with an energy expenditure of >7.2 METS were considered “vigorous” (e.g., activity with training effects when applied for a sufficient length of time and at an appropriate training frequency) [1].

**References**

[1] [Haskell WL](http://www.ncbi.nlm.nih.gov/pubmed?term=Haskell%20WL%5BAuthor%5D&cauthor=true&cauthor_uid=17671237), [Lee IM](http://www.ncbi.nlm.nih.gov/pubmed?term=Lee%20IM%5BAuthor%5D&cauthor=true&cauthor_uid=17671237), [Pate RR](http://www.ncbi.nlm.nih.gov/pubmed?term=Pate%20RR%5BAuthor%5D&cauthor=true&cauthor_uid=17671237), [Powell KE](http://www.ncbi.nlm.nih.gov/pubmed?term=Powell%20KE%5BAuthor%5D&cauthor=true&cauthor_uid=17671237), [Blair SN](http://www.ncbi.nlm.nih.gov/pubmed?term=Blair%20SN%5BAuthor%5D&cauthor=true&cauthor_uid=17671237), [Franklin BA](http://www.ncbi.nlm.nih.gov/pubmed?term=Franklin%20BA%5BAuthor%5D&cauthor=true&cauthor_uid=17671237), et al; [American College of Sports Medicine](http://www.ncbi.nlm.nih.gov/pubmed?term=American%20College%20of%20Sports%20Medicine%5BCorporate%20Author%5D); [American Heart Association](http://www.ncbi.nlm.nih.gov/pubmed?term=American%20Heart%20Association%5BCorporate%20Author%5D). [American College of Sports Medicine](http://www.ncbi.nlm.nih.gov/pubmed?term=American%20College%20of%20Sports%20Medicine%5BCorporate%20Author%5D); [American Heart Association](http://www.ncbi.nlm.nih.gov/pubmed?term=American%20Heart%20Association%5BCorporate%20Author%5D): Physical activity and public health: updated recommendation for adults from the American College of Sports Medicine and American Heart Association. Circulation. 2007;116:1081-93.
